# Tiaolipiwei Acupuncture Reduces Albuminuria by Alleviating Podocyte Lesions in a Rat Model of Diabetic Nephropathy

**DOI:** 10.1155/2018/1913691

**Published:** 2018-04-23

**Authors:** Zhilong Zhang, Xinju Li, Liang Liu, Jiya Sun, Xu Wang, Zhiheng Zhao, Yuanqing Yang

**Affiliations:** ^1^Tianjin Academy of Traditional Chinese Medicine Affiliated Hospital, Tianjin 300120, China; ^2^Tianjin University of Traditional Chinese Medicine, Tianjin 300193, China

## Abstract

**Background:**

Diabetic nephropathy is a common and serious complication of diabetes and a major cause of end-stage renal disease. Tiaolipiwei acupuncture is a safe treatment approach that may be effective for lowering albuminuria in diabetic nephropathy. Yet, the exact mechanisms of this therapeutic effect are unclear.

**Methods:**

A rodent model of type 2 diabetic nephropathy (T2DN) was induced by a high-fat diet combined with low-dose streptozotocin. T2DN rats were treated with Tiaolipiwei acupuncture (ACU) for 4, 8, or 12 weeks. At the end of treatment, urinary and blood samples were collected for analysis. Transmission electron microscopy was used to observe morphological changes, and protein expression levels of nephrin, CD2AP, podocalyxin, and desmin were quantified in renal tissue.

**Results:**

Compared to the T2DN groups, the T2DN + ACU groups showed significant improvements in 24-hour urinary protein, serum urea, cholesterol, and triglycerides at all time points. ACU treatment also improved the density of slit diaphragms. Simultaneously, ACU promoted the renal expression of nephrin, CD2AP, and podocalyxin and decreased the expression of desmin.

**Conclusion:**

Our study suggests that Tiaolipiwei acupuncture ameliorates podocyte lesions to reduce albuminuria and prevent the progression of T2DN in a rat model.

## 1. Introduction

Diabetic nephropathy (DN) is one of the most common and serious complications of diabetes and is a major cause of end-stage renal disease (ESRD) [1]. The estimated global prevalence of diabetes in 2017 was 8.8%, affecting more than 425 million adults worldwide. These figures are expected to increase by as much as 48% by the end of 2045, ultimately affecting more than 629 million adults [2]. Approximately 1 in 5 adults with type 2 diabetes has an estimated glomerular filtration rate (GFR) of <60 mL/min/1.73 m^2^, and between 30% and 50% of adults with diabetes exhibit elevated urinary albumin excretion [3]. These complications of diabetes place a heavy economic burden on families and society [4].

Hyperglycemia control, blood pressure control, and the use of angiotensin converting enzyme inhibitors or angiotensin receptor antagonists are standard treatment for patients with DN [5–7]. Yet, this regimen provides incomplete protection against renal failure, such that the prevalence of DN remains high [8]. Therefore, it is critical to develop more effective treatment strategies for patients with DN.

The appearance of microalbuminuria in patients with diabetes is an early marker of DN [9]. Microalbuminuria can develop into severe proteinuria and produce a progressive decline in GFR [10]. Accordingly, the control of albuminuria is an important goal of DN treatment. Clinical research has generally focused on the importance of podocyte injury in the initiation and progression of DN and albuminuria [11, 12]. Podocytes are terminally differentiated and highly specialized cells with foot processes that attach to the glomerular basement membrane (GBM) and are interconnected by a structure known as the slit diaphragm (SD). Podocyte injury has been described as one of the earliest pathological events in DN [13]. Because podocytes are not readily replaced, surviving podocytes employ various mechanisms to compensate for cell deficits [14, 15], most notably changing their size and shape to cover portions of the GBM left uncovered by podocyte loss. The best characterization of podocyte injury in DN involves reorganization of the actin cytoskeleton in foot processes, leading to foot process effacement and SD disruption [16, 17].

Acupuncture is an important part of complementary and alternative medicine in western countries and has been used for thousands of years in traditional Chinese medicine [18]. Accumulating evidence indicates that acupuncture is safe and has clinical efficacy in various disorders including cardiovascular diseases [19], chronic pain [20], functional constipation [21], and other pathological contexts [22, 23]. Acupuncture and acupuncture-like somatic nerve stimulation have also been successfully used to treat different kidney diseases and related complications [24] such as gouty renal damage [25] and renal disease progression [26]. The selection of appropriate acupoints is important to the therapeutic effects of acupuncture application. In a previous clinical trial, we demonstrated that Tiaolipiwei acupuncture, a specific acupoint prescription developed by Professor Zhang Zhilong, improved 24-hour urinary protein in patients with early-stage DN [27]. Yet, the exact mechanism by which Tiaolipiwei acupuncture lowers albuminuria is unclear.

In the present study, we established a rodent model of type 2 DN using a high-fat diet combined with low-dose intraperitoneal streptozotocin (STZ) [28, 29] and used this model to evaluate the mechanism by which Tiaolipiwei acupuncture attenuates albuminuria in a diabetic context.

## 2. Materials and Methods

### 2.1. Drugs and Reagents

Acupuncture needles were purchased from Suzhou Medical Sino-Foreign Joint Venture Suzhou Hua Tuo Medical Instruments Co., Ltd. STZ was purchased from Sigma-Aldrich Chemical Co. (MO, USA). Urine protein assay kits were purchased from Nanjing Jiancheng Bioengineering Institute (Nanjing, China). Anti-nephrin, anti-podocalyxin, and anti-desmin antibodies were purchased from Abcam Biotechnology (CA, USA). Anti-CD2AP antibody was purchased from R&D Biotechnology (MN, USA), and mouse anti-*β*-actin antibody was purchased from Boster Biotechnology (Wuhan, China). Horseradish peroxidase-conjugated goat anti-rabbit IgG and goat anti-mouse secondary antibodies were purchased from Boster Biotechnology (Wuhan, China). Rabbit anti-sheep IgG was purchased from EarthOx Life Sciences (CA, USA). Finally, a chemiluminescence kit was purchased from CWBIO (Beijing, China)

### 2.2. Animals

Male Sprague-Dawley rats (150–180 g) were obtained from the Experimental Animal Center of Tianjin Medical University. The research protocol was developed in accordance with guidelines for the care and use of animals and was approved by the animal ethics committee of Tianjin Medical University (approval number TMUaMEC2016008). Animals were housed in wire-bottom cages in a temperature- (24 ± 1°C) and humidity-controlled (~60%) room on a 12-hour light/dark cycle with free access to standard laboratory chow and tap water.

### 2.3. Experimental Design

All animals were allowed 7 days of adaptation prior to use in experiments. A high-fat diet and low-dose intraperitoneal STZ were used to establish the DN model. Briefly, rats were fed a high-fat diet (including 63.9% ordinary feed, 20% sucrose, 10% lard, 5% egg yolk powder, 1% cholesterol, and 0.1% pig bile sodium) for 8 weeks. At the end of this period, rats received a single intraperitoneal dose of STZ (40 mg/kg, freshly prepared in citrate buffer, pH 4.3). Blood glucose concentration was measured in tail blood using a reflectance meter (One Touch II, LifeScan Ltd., China) 72 h after the injection. Rats were excluded if the random blood glucose value was <16.7 mmol/L. Successful model induction was indicated by stability of an acceptable blood glucose level for more than 5 days; thereafter, blood glucose concentration was measured once per week. Rats were not given any hypoglycemic agents during the experimental period.

Diabetic rats were divided into six groups (*n* = 6 per group; average blood glucose levels were counterbalanced across the groups): DN 4 W (no treatment for 4 weeks), DN 8 W (no treatment for 8 weeks), DN 12 W (no treatment for 12 weeks), DN + ACU 4 W group (acupuncture treatment for 4 weeks), DN + ACU 8 W (acupuncture treatment for 8 weeks), and DN + ACU 12 W (acupuncture treatment for 12 weeks). Eighteen normal rats were used as a control group (no treatment) and divided into 3 groups (*n* = 6 per group): control 4 W, control 8 W, and control 12 W.

### 2.4. Acupuncture Strategy

Rats were placed in a supine position and fixed with binding clothes that did not interfere with spontaneous breathing. Acupuncture was performed at Zhongwan (CV12) and the bilateral Quchi (LI11), Hegu (LI4), Zusanli (ST36), Yinlingquan (SP9), Xuehai (SP10), Diji (SP8), Sanyinjiao (SP6), Fenglong (S40), and Taichong (LR3) acupoints as per the manual of acupuncture for experimental animals [30]. The needle is inserted vertically at a depth of 3-4 mm and maintained in position for a total of 30 min. Acupuncture was applied with the twirling method (120 times per min) and manipulation lasted for approximately 30 s. Acupuncture was applied once daily for 4, 8, or 12 weeks. All rats were sacrificed immediately after treatment.

### 2.5. Blood Sample and Tissue Collection

At the end of each treatment period, body weights were measured and rats were anesthetized by intraperitoneal injection of 10% chloral hydrate (3.5 mL/kg). The abdominal aorta was catheterized and used for blood sampling. Renal cortex tissues were fixed in a fixation fluid for transmission electron microscopy and the remaining kidney tissue was stored at −80°C until western blotting analysis. The levels of cholesterol (CHOL), triglycerides (TG), creatinine (Scr), and urea (Sur) in serum were determined using an automatic biochemical analyzer (GLAMOUR3000).

### 2.6. Urinary Albumin Excretion

Before sacrifice, rats were placed in metabolic cages for 24-hour urine collection to measure urinary albumin levels. Urine was collected and centrifuged, and aliquots of the supernatant were frozen at −80°C until analysis using urine protein assay kits (Jiancheng, Nanjing). The 24-hour urinary albumin excretion was calculated by multiplying the urinary albumin excretion by the 24-hour urine volume.

### 2.7. Renal Histology

After sacrifice and abdominal cavity blood sampling, the bilateral kidneys were excised and the renal cortex was fixed in 3% glutaraldehyde, postfixed in 1% osmium tetroxide, imbued with uranyl acetate, and embedded in epoxy resin (Epon). Specimens were then thin-sectioned and examined under a transmission electron microscope (JEM- 1200EX II; JEOL, Tokyo, Japan).

Electron micrographs of 5–10 glomeruli per kidney were randomly taken at magnifications of 10,000x and 20,0000x for each rat. Mean thickness of the GBM was calculated using measurements from 3 different sites in the cross section with ImageJ software (National Institutes of Health, MD, USA). Tangentially sectioned GBM was excluded from the analysis. Photomicrographs of the GBM were also analyzed for the density of slit pores between podocyte foot processes as previously described [31, 32]. Briefly, the number of slit pores was counted and divided by the GBM length (mm) to yield a linear density.

### 2.8. Protein Extraction and Western Blotting

Total protein in renal cortex tissues was extracted and protein concentrations were measured using a BCA kit. Proteins were separated by SDS-PAGE and transferred onto polyvinylidene fluoride membranes (Bio-Rad Laboratories, Inc.). Membranes were blocked with 5% nonfat milk in Tris-buffered saline containing Tween 20 (0.05%) for 1 h at room temperature and then incubated with primary antibody overnight at 4°C. After incubation with a horseradish peroxidase-conjugated secondary antibody, immunoreactive bands were observed using ECL substrate as per the manufacturer's protocol. *β*-Actin antibody was used as a loading control. A densitometric analysis was performed using ImageJ software.

### 2.9. Statistical Analysis

Data are expressed as the mean ± standard error of the mean. One-way analysis of variance and Tukey's post hoc pairwise comparisons were performed using R software. Differences were considered to be statistically significant if the *P* value was <0.05.

## 3. Results

### 3.1. Physiological and Biochemical Indicators

**Figure 1:**
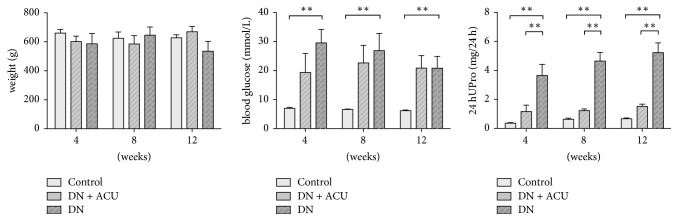
Body weight, blood glucose, and 24-hour urine protein in each group. ACU: acupuncture; DN: diabetic nephropathy model; UPro: urine protein. *∗∗* indicates *P* < 0.05.

**Figure 2:**
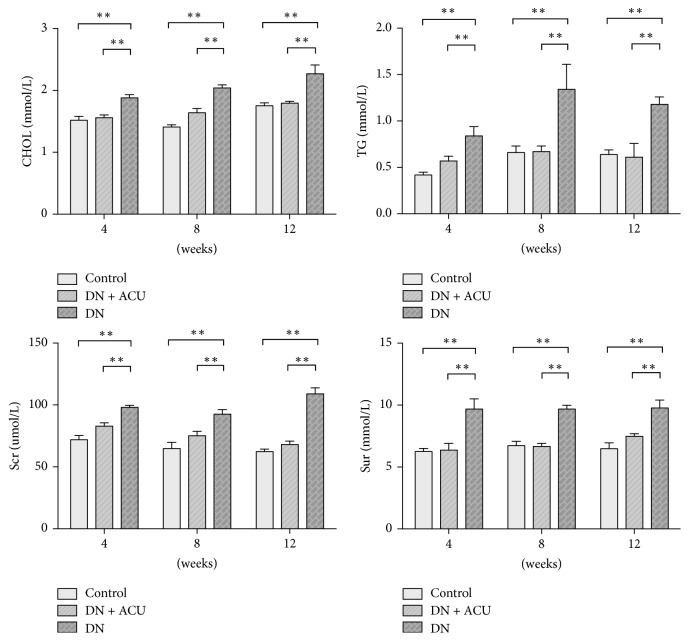
Markers of kidney function and blood lipids in each group. ACU: acupuncture; CHOL: cholesterol; DN: diabetic nephropathy model; Scr: serum creatinine; Sur: serum urea; TG: triglycerides. *∗∗* indicates *P* < 0.05.

Values of BG, 24-hour urinary albumin, Sur, Scr, CHOL, and TG were significantly higher in the DN groups than in the control groups (all *P* < 0.05). In contrast, body weights did not significantly vary among all groups. Groups that received acupuncture treatment (DN + ACU) had significantly lower values of 24-hour urinary albumin, CHOL, TG, Scr, and Sur than the DN groups did, while there was no significant effect of acupuncture on BG (Figures 1 and 2).

### 3.2. Effects of Tiaolipiwei Acupuncture on Renal Tissue Morphology

**Figure 3:**
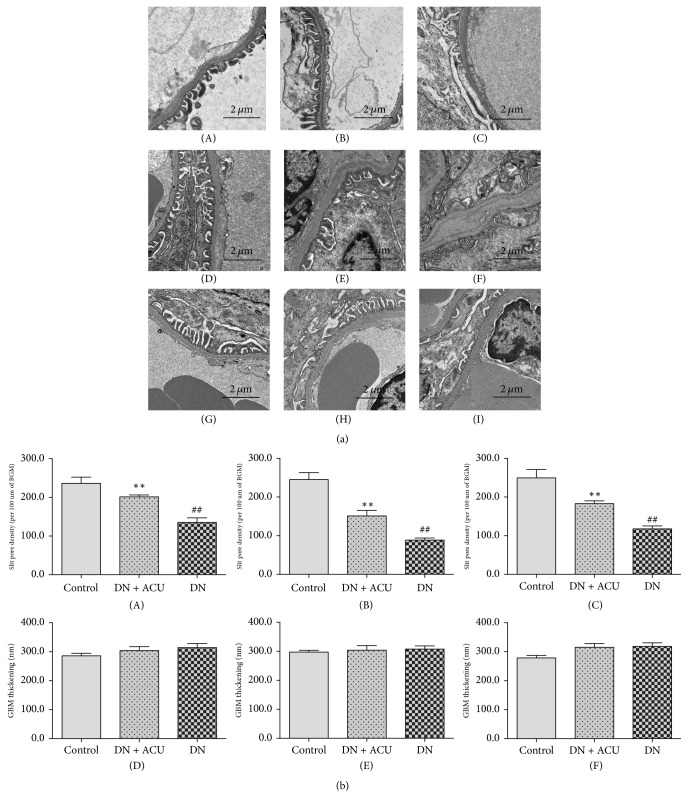
(a) Ultrastructural features of the renal cortex observed under transmission electron microscopy in all groups. (A) Control 4 W, (B) DN + ACU 4 W, (C) DN 4 W, (D) Control 8 W, (E) DN + ACU 8 W, (F) DN 8 W, (G) Control 12 W, (H) DN + ACU 12 W, and (I) DN 12 W. (b) The number of slit pores between podocyte foot processes and glomerular basement membrane thickness at 4 weeks after treatment (A, D), 8 weeks after treatment (B, E), and 12 weeks after treatment (C, F). ACU: acupuncture; DN: diabetic nephropathy model; GBM: glomerular basement membrane. *∗∗* indicates *P* < 0.05 versus DN groups; ## indicates *P* < 0.05 versus control groups.

**Figure 4:**
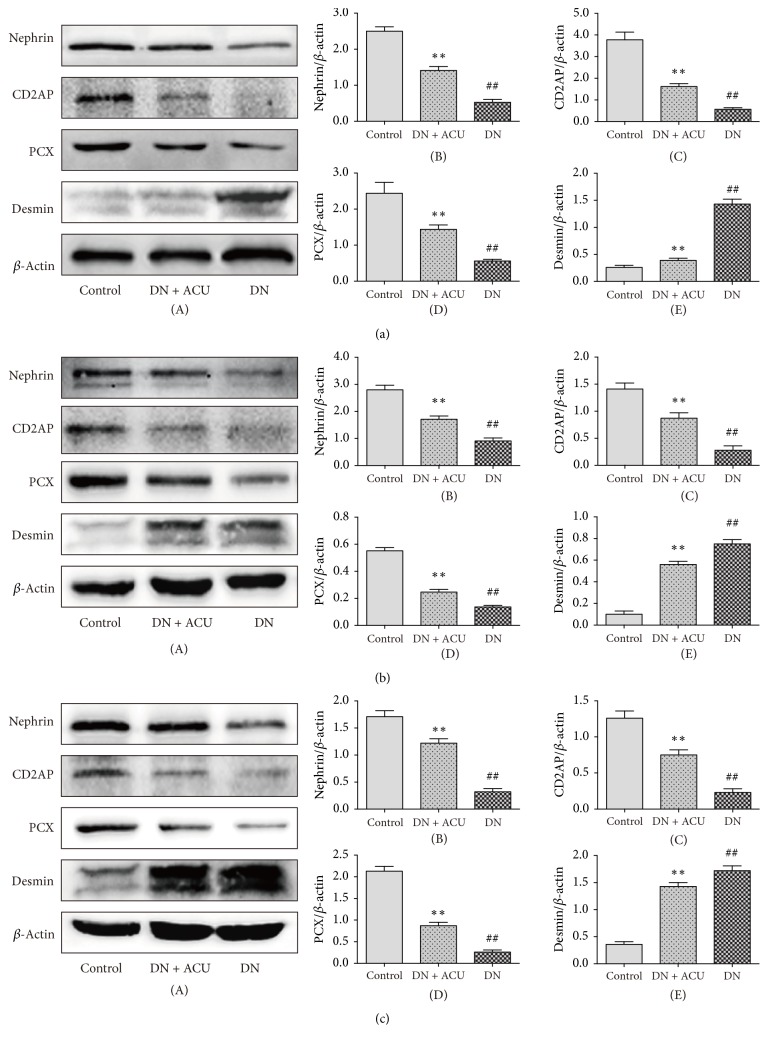
Protein expression in the renal cortex in all groups. (a) After 4 weeks of treatment, (b) after 8 weeks of treatment, and (c) after 12 weeks of treatment. *β*-Actin was used for the protein loading control. ACU: acupuncture; DN: diabetic nephropathy model; PCX: podocalyxin. *∗∗* indicates *P* < 0.05 versus DN groups; ## indicates *P* < 0.05 versus control groups.

Given the role of podocyte lesions in DN-associated albuminuria, we examined renal morphology in treated and untreated rats using transmission electron microscopy. Ultrastructural observation of renal cortical tissue from control group rats revealed normal GBMs without foot process fusion (Figure 3(a), A, D, and G). In DN group rats, some podocyte foot processes exhibited fusion or complete destruction or disappearance. The GBM showed thickening in these animals, although the effect was nonsignificant compared to the control group (Figure 3(a), B, E, and H). In contrast, podocyte injury showed attenuation in DN + ACU group animals (Figure 3(a), C, F, and I). Animals that received Tiaolipiwei acupuncture showed improved SD density (Figure 4(b), A, B, and C) while GBM thickness showed variable improvement.

### 3.3. Effects of Tiaolipiwei Acupuncture on Renal Nephrin, CD2AP, Desmin, and Podocalyxin Expression

We next examined the renal expression of podocyte proteins involved in slit diaphragm regulation. Compared to the control groups, all DN groups exhibited a decreased expression of nephrin, CD2AP, and podocalyxin and increased expression of desmin. In contrast, normal patterns of protein expression were at least partly recovered in all DN + ACU groups (Figure 4).

## 4. Discussion

The present study used a rat model of type 2 DN induced by a high-fat diet and STZ injection to examine the efficacy and therapeutic mechanisms of Tiaolipiwei acupuncture in DN. Specifically, acupuncture treatment restored SD density, decreased albuminuria, and improved blood lipid concentrations. It also upregulated the expression of SD proteins (nephrin and CD2AP) and the apical membrane protein podocalyxin and downregulated the cytoskeletal intermediate filament protein desmin. Accordingly, we conclude that all treatment durations (4–12 weeks) of Tiaolipiwei acupuncture protected against podocyte injury as a component of its therapeutic effect on DN albuminuria.

Given the limited effects of standard treatments on DN progression, several new agents such as empagliflozin have been developed to more effectively reduce proteinuria; yet, these agents are also associated with side effects such as urinary tract infection, hypovolemia, and ketoacidosis [33]. For this reason, traditional Chinese medicine offers a significant advantage and utility for the management of DN. Despite a lack of high-quality evidence, several studies have indicated that acupuncture is effective for reducing albuminuria in patients with DN [27]. Our present results add to this data by demonstrating the therapeutic efficacy of a specific form of acupuncture, Tiaolipiwei acupuncture, in a rodent model. After at least 4 weeks of daily treatment, acupuncture reduced albuminuria, improved indicators of renal function, and regulated blood lipids in DN model rats. These findings inform the ability of continuous acupuncture to produce sustained effects in DN.

Podocytes are the last barrier between the renal artery and the primary urine and are the first major site of damage in DN. As a result, these cells play an important role in albuminuria. Podocytes are terminally differentiated and unable to replicate or significantly regenerate in adults, such that podocyte loss or injury is an irreversible step in disease development [34]. SDs between podocyte foot processes are composed of multiple protein complexes and contribute significantly to the integrity of the glomerular filtration barrier. In the present study, we found that acupuncture protected SD density, indicating that acupuncture exerted a protective effect on podocytes. The increase in GBM thickness was not significant in our study, although relevant previous studies have demonstrated that accumulation of collagen type IV can thicken the GBM in STZ-treated rodent models of diabetes [35, 36]. This observation in the present study may have been related to the termination of the high-fat diet after successful model establishment, resulting in only moderately severe lesions and thus no marked changes in GBM thickness.

Several animal studies have convincingly demonstrated that key signaling alterations in podocytes are sufficient to affect the course of diabetic albuminuria development [37–39]. In this study, we found that acupuncture had effects on podocyte protein expression.

Proteins including nephrin, CD2AP, podocalyxin, and desmin play pivotal roles in maintaining podocyte integrity. Nephrin is a major transmembrane protein located in the glomerular SD region [40, 41] that regulates glomerular filtration function and thus the extent of proteinuria in DN [42]. Injection of an anti-nephrin antibody induces substructural alterations of the SD in animals, yielding reductions in permselectivity and consequently proteinuria [43]. CD2AP is another transmembrane protein that interacts with nephrin to maintain the function of the cytoskeleton and SD. Damage to CD2AP leads to disruption of the podocyte cytoskeleton and massive proteinuria [44, 45]. In this study, the ability of Tiaolipiwei acupuncture to sustain expression levels of nephrin and CD2AP may represent a critical component of its therapeutic mechanism.

The apical surface of podocytes facing the urinary space is coated with a sialic acid-rich glycocalyx known as the epithelial polyanion and is mainly composed of podocalyxin that contributes to the negative charge of the glomerular membrane [46, 47]. In our study, podocalyxin expression was also increased by acupuncture, suggesting that the therapeutic mechanism may have involved protection of the podocyte apical surface. Desmin, an intermediate filament protein, is also regulated in response to podocyte injury. Zou et al. [48] showed that the upregulation of desmin increased the mechanical stability of podocytes to enable morphological changes on the tensile glomerular capillary wall. Our finding of increased desmin expression in DN rats and decreased expression in DN rats after Tiaolipiwei acupuncture treatment suggests that acupuncture prevented DN-associated morphological alterations in podocytes. Future research is necessary to determine whether alterations in desmin were direct or indirect effects of treatment.

Additionally, several studies have shown that the accumulation of cholesterol in podocytes can contribute to podocyte damage and accelerate the progression of kidney disease [49]. In this study, the Tiaolipiwei acupuncture significantly reduced the levels of blood lipids in DN rats. This may be an alternative explanation for the observed effects of acupuncture on podocyte injury and albuminuria.

Interestingly, we detected a slight decrease in blood glucose level in the DN + ACU groups compared to the DN groups, although this difference was not statistically significant. Previous studies have suggested that acupuncture can alter blood glucose level in the early stages of diabetes [50]. Further, a few other studies have shown that protecting podocytes from hyperglycemia with a podocyte-specific deletion of the glucose transporter solute carrier family 2 or facilitated glucose transporter member 4 (SLC2A4, also known as GLUT4) [51] or from the resulting oxidative stress [12] can prevent diabetes-associated albuminuria without restoring normal levels of glucose. The underlying mechanism of the effect of Tiaolipiwei acupuncture on DN observed in the present study may also be similar, and further studies are needed to confirm this.

Complex relationships can exist among specific acupoints (e.g., synergy and antagonism) in the generation of a therapeutic effect [52–54]. In clinical settings, acupuncturists select acupoints in accordance with the principles of traditional Chinese medicine and the experiences of renowned doctors. In this study, the acupoints used in Tiaolipiwei acupuncture were derived from the experience of Professor Zhang Zhilong, a recognized national expert in acupuncture, and are effective against diabetes and related complications [27, 55–57].

Our previous article [58] explained the TCM theoretical mechanism on diabetic nephropathy caused by the disorders of ascending and descending in the spleen and stomach. Overeating high-saturated-fat and high-sugar food leads to deficiency of the spleen and stomach and then the physiological dysfunction of ascending and descending in the spleen and stomach. According to TCM theory, “ascending” represents the utilization and distribution of human nutrition and “descending” represents the normal excretion of metabolic waste. The spleen is in charge of ascending, while the stomach is responsible for descending, and they play decisive roles in the ascending and descending function in the human body. With long-term ascending and descending function disorders, nutrients cannot be used and excreted due to proteinuria, and metabolic waste cannot be excreted normally, which results in the accumulation of oxidation products. This process leads to the deficiency of the kidney because it cannot be nourished, and this further aggravates the proteinuria.

Thus, Tiaolipiwei acupuncture is adopted according to the etiology. The main acupoints that we choose for regulating ascending and descending are Zhongwan (CV12) and Zusanli (ST36); for replenishing qi and blood of the spleen and stomach are Xuehai (SP10) and Diji (SP8); for ascending function are Sanyinjiao (SP6) and Yinlingquan (SP9); for descending function are Quchi (LI11), Hegu (LI4), Fenglong (S40), and Taichong (LR3).

A recent study [59] has found that this needling method can improve the state of oxidative stress in patients and can regulate the ascending and descending of the spleen and stomach to a certain extent. Moreover, numerous studies [12, 60] have shown that oxidative stress is closely related to podocyte injury. This may likely be the mechanism by which Tiaolipiwei acupuncture reduces proteinuria in DN; however, the exact biological mechanism behind this effect is unclear. Studies in rats can provide insights into the therapeutic mechanism of Tiaolipiwei acupuncture in human clinical cases.

The present study has limitations as follows. First, the current therapeutic understanding of acupuncture is that it affects regulatory functions on a body-wide scale [61] that in turn affect the target organ. This is a three-step process involving local correspondence, function transmission, and target organ activation [62]. In the present study, we only investigated the processes related to the target organ, so additional studies are needed to address the effects of acupuncture on the first 2 steps, that is, local correspondence and function transmission. Second, the present study focused only on the critical proteins of the podocyte and did not assess the signaling pathways involved in repairing podocyte injuries such as the Notch signaling pathway, which has been proven to be related to both acupuncture [63] and podocyte injuries [64]. Third, the role of autophagy in podocyte injuries, an important topic, was not investigated in the present study. Autophagy is a natural, regulated, destructive mechanism of the cell that disassembles unnecessary or dysfunctional components that play an important role in the repair of injured podocytes, which are terminally differentiated cells that are unable to replicate or significantly regenerate in adults. Studies have shown that autophagy administration can repair podocyte injuries and reduce significant proteinuria [65]. In contrast, increasing evidence suggests that acupuncture has therapeutic effects in disease contexts involving autophagy pathology [66]. For this reason, future studies should investigate the effects of acupuncture on autophagy to determine whether therapeutic effects are direct effects or indirect consequences of autophagy modification.

## 5. Conclusion

In conclusion, our study suggests that Tiaolipiwei acupuncture ameliorates podocyte lesions to reduce albuminuria and prevent DN progression in a rodent model.

## Data Availability

All the data involved in this paper are available to all the readers. Thus, the readers can access the data in this paper or contact the corresponding author by e-mail.
